# Recent Trend in Artificial Intelligence-Assisted Biomedical Publishing: A Quantitative Bibliometric Analysis

**DOI:** 10.7759/cureus.39224

**Published:** 2023-05-19

**Authors:** Larry E Miller, Debjani Bhattacharyya, Valerie M Miller, Mehul Bhattacharyya

**Affiliations:** 1 Clinical Research, Miller Scientific, Johnson City, USA; 2 Education, University of Massachusetts Lowell, Lowell, USA; 3 Leadership, University of the Cumberlands, Williamsburg, USA

**Keywords:** references, machine learning, large language model, chatgpt, artificial intelligence

## Abstract

The rapid advancements in artificial intelligence (AI) technology in recent years have led to its integration into biomedical publishing. However, the extent to which AI has contributed to developing biomedical literature is unclear. This study aimed to identify trends in AI-generated content within peer-reviewed biomedical literature. We first tested the sensitivity and specificity of commercially available AI-detection software (Originality.AI, Collingwood, Ontario, Canada). Next, we conducted a MEDLINE (Medical Literature Analysis and Retrieval System Online) search to identify randomized controlled trials with available abstracts indexed between January 2020 and March 2023. We randomly selected 30 abstracts per quarter during this period and pasted the abstracts into the AI detection software to determine the probability of AI-generated content. The software yielded 100% sensitivity, 95% specificity, and excellent overall discriminatory ability with an area under the receiving operating curve of 97.6%. Among the 390 MEDLINE-indexed abstracts included in the analysis, the prevalence with a high probability (≥ 90%) of AI-generated text increased during the study period from 21.7% to 36.7% (p=0.01) based on a chi-square test for trend. The increasing prevalence of AI-generated text during the study period was also observed in various sensitivity analyses using AI probability thresholds ranging from 50% to 99% (all p≤0.01). The results of this study suggest that the prevalence of AI-assisted publishing in peer-reviewed journals has been increasing in recent years, even before the widespread adoption of ChatGPT (OpenAI, San Francisco, California, United States) and similar tools. The extent to which natural writing characteristics of the authors, utilization of common AI-powered applications, and introduction of AI elements during the post-acceptance publication phase influence AI detection scores warrants further study.

## Introduction and background

The rapid advancements in artificial intelligence (AI) technology in recent years have led to its integration into many areas of medicine such as drug discovery [1], biomedical imaging [2], clinical decision-making [3], and biomedical publishing [4,5]. Potential applications of AI in biomedical publishing include improving text clarity, AI-assisted peer review [6,7], and detection of AI-assisted writing [8].

While public utilization of AI has been limited historically, the availability of the large language model Generative Pre-trained Transformer-3 (GPT-3, OpenAI, San Francisco, California, United States) in July 2020 and Chat Generative Pre-trained Transformer (ChatGPT, OpenAI) [9] in November 2022 has led to widespread adoption of AI, with ChatGPT gaining over one billion monthly users by March 2023 [10]. ChatGPT uses the Transformer natural language processing framework to interpret complex linguistic patterns. ChatGPT has acquired a comprehensive understanding of human communication through extensive training on large language datasets. This training enables ChatGPT to generate responses that are contextually appropriate and engage in intelligent, human-like conversations.

In parallel with the increasing utilization of generative AI, software purporting high accuracy in detecting AI-generated content is now widely available. Although the specific algorithms used by these tools are proprietary, they typically evaluate writing characteristics such as text perplexity and burstiness. Notably, humans tend to write with high degrees of these characteristics, whereas AI-generated writing reflects lower levels of perplexity and burstiness [11]. These detectors use natural language processing techniques to analyze and understand text, and deep learning techniques to recognize patterns and features within the text [12]. Ultimately, this allows the detectors to distinguish AI-generated content from human-generated content.

The extent to which this widespread availability of ChatGPT and other AI-assisted writing tools has contributed to developing biomedical literature is unclear. Thus the purpose of this study was to identify recent trends in AI-generated content within peer-reviewed biomedical literature.

## Review

We conducted a quantitative bibliographic analysis to identify recent trends in AI-developed content within peer-reviewed biomedical literature. 

AI-detection software sensitivity and specificity testing

The AI-detection software used in this study, Originality.AI (Collingwood, Ontario, Canada), was selected based on its claimed superior accuracy in detecting AI-generated content compared to other AI detectors [12]. First, we tested software sensitivity, which is the ability to correctly detect AI-generated text. We generated 60 abstracts with ChatGPT using the following prompt variations:

*Please write a 250 to 300-word abstract for a peer-reviewed journal specializing in* (oncology, cardiology, neurology, immunology, endocrinology, infectious disease, psychiatry, respiratory medicine, gastroenterology, or nephrology) *that reports the results of a randomized controlled trial where outcomes in the investigational group were* (better than, worse than, or similar to) *those in the control group. Write the abstract using* (unstructured, or background, methods, results, conclusions) *format.*

**Figure 1:**
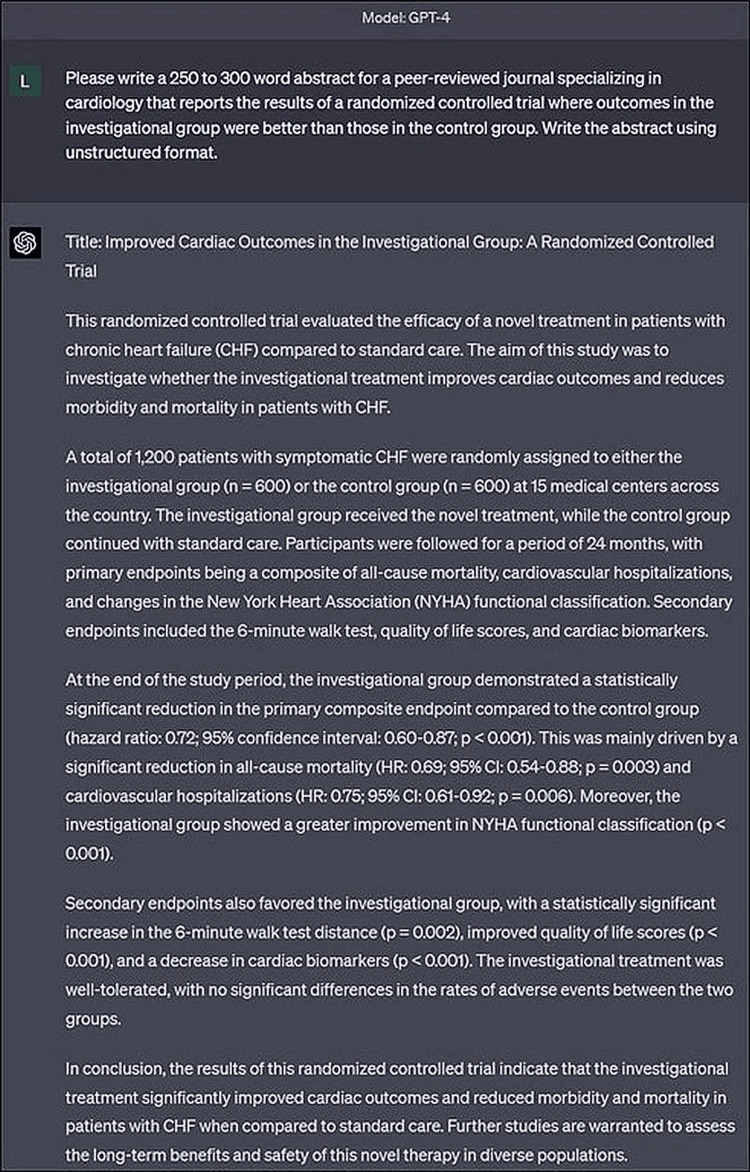
Sample ChatGPT-generated abstract developed during sensitivity testing of artificial intelligence detection software.

Overall, we issued 60 unique commands covering the 10 therapeutic areas, three clinical scenarios, and two abstract formats listed above, where each command was entered in a new chat window to minimize redundant answers that may be associated with previous conversations. An example of an abstract generated by ChatGPT (Model GPT-4) during the sensitivity testing is provided in Figure 1.

We next tested software specificity, which is the ability of the software to correctly identify human-generated text. We randomly selected 60 randomized controlled trials with available abstracts indexed in MEDLINE (Medical Literature Analysis and Retrieval System Online) between January 1980 and December 1989, a period with negligible AI utilization. We pasted the abstract from each paper into the AI detection software, which reported the probability of AI-generated content on a scale ranging from 0% to 100%.

Literature search and AI-detection testing

We subsequently conducted a MEDLINE search to identify randomized controlled trials with available abstracts indexed between January 2020 and March 2023. The MEDLINE search used the following string: “2020/01/01:2023/03/31[Date - Entry] AND randomizedcontrolledtrial[Filter] AND fha[Filter]”. Among all eligible abstracts in the study period, we used a random number generator to randomly selected 30 abstracts per quarter, each containing at least 50 words based on the minimum requirements of the AI-detection software. As before, we pasted the abstracts into the AI-detection software to determine the probability of AI-generated content.

Data analysis

Assuming that 15% of abstracts in 2020 were AI-generated and that the prevalence would increase by 7.5% annually, the study had 89% statistical power using a two-sided Z test and a significance level of 0.05. We used the chi-square test for trend to test the hypothesis that AI-generated abstracts were becoming more prevalent over time. Abstracts that received a score ≥90% were deemed to be AI-generated based on the recommendations of the software manufacturer. We tested the robustness of this threshold by performing sensitivity analysis using thresholds of 50%, 75%, 95%, and 99%.

Results

Among the 60 abstracts generated by ChatGPT, all were scored with a 100% probability of being AI-generated. Among the 60 abstracts that were MEDLINE-indexed in the 1980s, the median probability of AI generation was 8% (interquartile range = 3-19%), with 57 of 60 receiving a score less than 90%. Ultimately, these results yielded 100 sensitivity, 95% specificity, and excellent overall accuracy with an area under the receiving operating curve of 97.6% (standard error = 1.6%).

**Figure 2:**
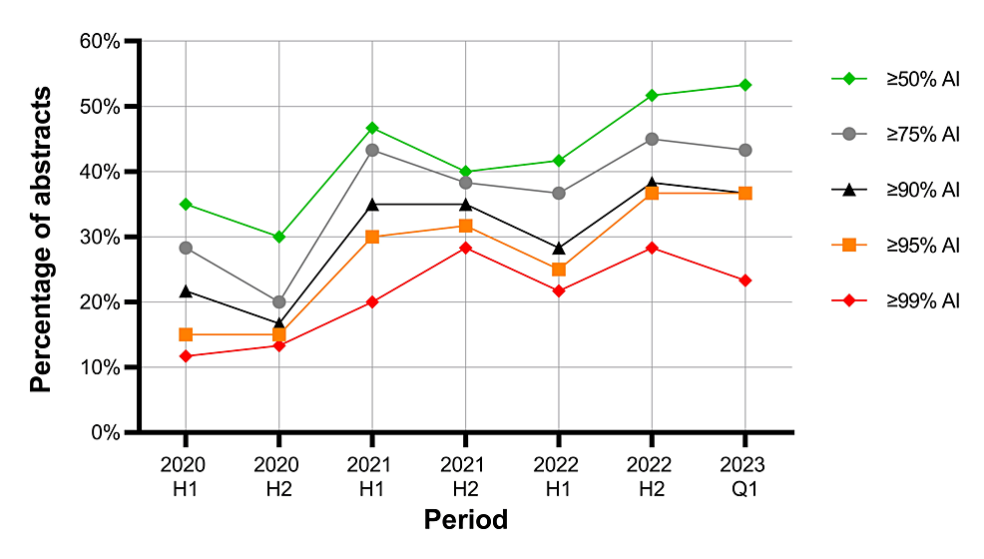
Trends in published abstracts by the predicted probability of artificial intelligence (AI)-generated text. Between the first half of 2020 and the first quarter of 2023, the percentage of abstracts with ≥90% predicted probability of AI-generated text increased from 21.7% to 36.7% (p=0.01). Using other detection thresholds, the percentage of abstracts with AI probability ≥99% (11.7% to 23.3%; p=0.01), ≥95% (15.0% to 36.7%; p=0.001), ≥75% (28.3% to 43.3%; p=0.01), and ≥50% (35.0% to 53.3%; p=0.01) also increased during this period.

Among the 72,941 results identified in the MEDLINE search spanning 2020 to 2023, 390 abstracts were included in the analysis. The prevalence of abstracts with a high probability (≥ 90%) of AI-generated text increased during the study period, from 21.7% to 36.7% (p=0.01). The increasing prevalence of AI-generated text during the study period was also observed in each sensitivity analysis (all p≤0.01) (Figure 2).

Discussion

This quantitative bibliographic analysis of randomized controlled trials published in peer-reviewed journals suggests a rising trend in AI-assisted biomedical writing in the years preceding the widespread adoption of ChatGPT. Several journals have updated their author guidelines to include requirements for AI-assistance disclosures [13], and some groups have published position statements regarding AI authorship and its use in manuscript development [14,15]. Despite this growing awareness, there is a lack of studies describing AI-utilization trends in the biomedical literature. The results of this study provide an initial framework for developing definitions and thresholds for detecting and defining AI-assisted writing.

A fundamental question raised by this study is how rates of AI-assisted writing have been increasing before the widespread availability of ChatGPT and similar tools. Likely, most authors of biomedical literature have unknowingly used AI technology for years. In fact, the authors of this paper used AI applications to assist with manuscript development (see the Acknowledgements section for details). Typical AI-powered writing assistance applications such as the Editor feature in Word (Microsoft Corporation, Redmond, Washington, United States) [16], Smart Compose in Google Docs (Google LLC, Mountain View, California, United States) [17], and Grammarly (Grammarly Inc., San Francisco, California, United States) [18] are widely used without criticism regarding their use in the writing process. Even in papers developed without AI assistance, journal copyeditors may subsequently revise the article using AI in their workflows before publication [19,20]. It is therefore not surprising that some papers in the pre-ChatGPT era were flagged with AI-generated content. The extent to which natural writing characteristics of the authors, utilization of common AI-powered applications, and introduction of AI elements during the post-acceptance publication phase influence AI detection scores warrants further study. 

The current state of biomedical publishing is characterized by a significant transition due to rapid advancement in AI technology. Although some journals and societies are starting to acknowledge and define the ethical considerations involved in AI-assisted publishing [21], there remains much inconsistency in addressing these concerns, with most journals failing to specify AI-related authorship guidelines. Inevitably, AI-assisted writing will likely continue to infiltrate the biomedical literature, although human judgment should always prevail [22]. In support of this statement, Microsoft is currently conducting pilot testing on an AI-assisted writing feature called “Co-pilot” [23]. Once fully released, this software will become an integral part of Word and will likely be widely utilized when developing biomedical papers. Instead of attempting to impede the use of AI in biomedical writing, efforts should focus on harnessing the powers of AI to enhance clinical research and writing efficiency while promoting transparency in its use to uphold ethical standards. To achieve this, there is a need for uniform and widely adopted standards to establish comprehensive guidelines addressing critical issues like transparency, disclosure, and responsible usage of AI in biomedical writing, a sentiment echoed by others [24,25].

Despite the novelty of this study’s research question, this work has several limitations. We only analyzed the abstracts from randomized controlled trials indexed in MEDLINE. While this provided some standardization in abstract content and access to text without cost, we did not examine the full text of papers or abstracts with different study designs. Therefore, the generalizability of the trends observed in this study may be limited. Future studies could address this limitation by analyzing the same trends associated with different study designs or by analyzing the full text of manuscripts as opposed to only abstracts. Specifically, examining the individual components of a manuscript such as the abstract, introduction, methods, and other sections could identify the most common locations of AI-generated content within papers. A second limitation of the study was that we used a single AI detector in this study. While the sensitivity and specificity of the detector were excellent, other AI-detection software programs are available (Table 1) that might yield different results, which could be a topic for future research. Finally, it is difficult to attribute the use of AI within a published work to a specific person or group since published abstracts and manuscripts may reflect the combined inputs from authors, peer reviewers, journal editors, fee-based language services, and copyeditors. Future evaluations of this topic are possible but would require collaboration with publishers to gain access to the required content and metrics.

**Table 1:** Selected publicly available software for detecting artificial intelligence-generated text.

Software	Website
Fee-Based	
Originality.ai	https://originality.ai/
Copyleaks	https://copyleaks.com/
Crossplag	https://crossplag.com/ai-content-detector/
Writer	https://writer.com/
Free To Use	
GPT-2 Output Detector	https://openai-openai-detector.hf.space/
GPT Zero	https://gptzero.me/

## Conclusions

This bibliographic analysis of biomedical literature demonstrated that the prevalence of AI-assisted writing in peer-reviewed journals has been increasing in recent years, even before the widespread adoption of ChatGPT. These findings highlight the growing integration of AI technology into the writing process, a trend that is anticipated to continue in the future. Given the recent heightened scrutiny surrounding ChatGPT and its related applications, we must recognize that AI is already deeply embedded within many commonly used writing tools, perhaps unknowingly to many users. The development of guidelines to promote the responsible and ethical use of AI technology when developing biomedical literature is warranted.
